# Egg Size Scales Negatively With System Size in a Periodic Fish Species

**DOI:** 10.1002/ece3.70426

**Published:** 2024-10-17

**Authors:** Scott T. Koenigbauer, Zachary S. Feiner, Benjamin Dickinson, Stephanie L. Shaw, L. Zoe Almeida, Mark R. DuFour, Alexander J. Gatch, Claire Schraidt, Tomas O. Höök

**Affiliations:** ^1^ Department of Forestry and Natural Resources Purdue University West Lafayette Indiana USA; ^2^ Office of Applied Science, Wisconsin Department of Natural Resources Science Operations Center Madison Wisconsin USA; ^3^ Center for Limnology University of Wisconsin‐Madison Madison Wisconsin USA; ^4^ Indiana Department of Natural Resources Michigan Indiana USA; ^5^ Office of Applied Science, Wisconsin Department of Natural Resources Escanaba Lake Research Station Madison Wisconsin USA; ^6^ Cornell University Bridgeport New York USA; ^7^ Lake Erie Biological Station, Great Lakes Science Center United States Geological Survey Huron Ohio USA; ^8^ Tunison Laboratory of Aquatic Science, Great Lakes Science Center United States Geological Survey Cortland New York USA; ^9^ Osborn Memorial Laboratories, Department of Ecology and Evolutionary Biology Yale University New Haven Connecticut USA; ^10^ Illinois‐Indiana Sea Grant West Lafayette Indiana USA

**Keywords:** freshwater fish, Great Lakes, inland lakes, maternal effects, reproduction, yellow perch

## Abstract

Optimal egg size theory implies that female organisms balance between fecundity and individual offspring investment according to their environment. Past interspecific studies suggest that fishes in large marine systems generally produce smaller eggs than those in small freshwater systems. We tested whether intraspecific egg size variation reflected a similar pattern by comparing egg size among yellow perch (*Perca flavescens*) populations inhabiting a range of system sizes. In 2018, 2019, and 2023, we collected yellow perch egg samples from 12 locations in systems ranging in surface area from 37 to 5,390,492 ha. First, we found that egg diameter significantly increased with maternal total length in five of eight individually tested populations. After accounting for these maternal effects, we found a significant interaction, where females inhabiting larger lakes, such as the main basins of Lakes Erie and Michigan, produced smaller eggs than those in smaller inland lakes, and the greatest differences were demonstrated among females of greater total length. This egg size variation in the largest females is consistent with interspecific egg size comparisons between marine and freshwater fishes. However, by examining a single species across vastly different environments, we were able to support theoretical expectations that maternal investment in offspring should vary with environmental conditions controlling early‐life resource acquisition and competition.

## Graphical abstract

Past studies have found freshwater fishes produce larger eggs on average than marine fishes. However, such comparisons may be complicated by life history and morphological variation among species. In this study, yellow perch egg size was compared among populations in systems ranging six orders of magnitude in surface area to test the relationship between egg size and system size within a single species.

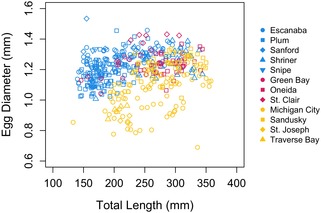

## 1 Introduction

Egg size and its implications for offspring survival and population recruitment have long been studied in fish. Females that produce larger eggs provision their offspring for larger size‐at‐hatch, which may result in reduced risk of starvation, faster growth, increased foraging ability, and improved predator avoidance (Duarte and Alcaraz 1989; Einum and Fleming 2000). However, the duration of egg incubation can further contribute to variation in hatching size (e.g., size‐at‐hatch can increase with incubation duration; Pauly and Pullin 1988). Increasing egg size is consistently positively correlated with increasing offspring size and often positively correlated with increasing survival in early life (Koenigbauer and Höök 2023). Therefore, offspring hatched from larger eggs could ultimately experience higher survival to recruitment relative to offspring from smaller eggs due to their expedited advancement through critical post‐hatch ontogenetic stages and relatively lower instantaneous mortality rates (Berkeley, Chapman, and Sogard 2004). Although offspring may experience early life benefits from larger egg size, females must compromise between egg size and fecundity (Einum and Fleming 2000). That is, females are limited in ovary volume and reproductive energy available for gonadal investment, which implies that producing larger eggs will result in a cost related to additional fecundity (Kamler 2005). Based on this trade‐off, the theory of optimal egg size suggests that selection leads to adaptive variation in egg size to maximize total surviving offspring production according to environmental conditions (Smith and Fretwell 1974; Einum and Fleming 2002).

Optimal egg size is known to differ across fish species and systems. For example, comparisons of egg size have been made between fishes from large marine systems to those in small freshwater systems, but these comparisons have generally been interspecific and have not considered large freshwater systems (Duarte and Alcaraz 1989; Einum and Fleming 2002). Duarte and Alcaraz (1989) compared mean egg sizes of 51 marine and 46 freshwater fish species with varying life history strategies and found that freshwater species produce larger eggs on average. Houde (1994) suggested that freshwater and marine fishes experience different selective environments during early life, leading these groups to generally produce different‐sized offspring. In small freshwater systems, recruitment seems more likely to be strongly influenced during the later juvenile stage, due to density‐dependent factors such as competition for prey resources (e.g., Feiner, Shaw, and Sass 2019). In contrast, larvae inhabiting large, stochastic marine systems often emerge at relatively smaller sizes, and recruitment success is more likely determined in the early larval stage, based upon whether individual larvae experience favorable or unfavorable conditions in highly variable environments (Cushing 1990; Chambers 1997; Ludsin, DeVanna, and Smith 2014). While Houde's (1994) theory of differential reproductive ecology associated with system size is based on larval size‐at‐hatch, it has implications for egg size as well, because egg size is correlated with size‐at‐hatch (Pepin, Orr, and Anderson 1997; Nissling et al. 1998; Rideout, Trippel, and Litvak 2005). Further, studies of marine fishes' reproductive strategy suggest females either hedge their bets with within‐clutch egg size variation (e.g., Marshall, Bonduriansky, and Bussière 2008) or produce many small eggs in systems characterized by Sweepstakes reproductive success (Hedgecock and Pudovkin 2011). Thus, females in large systems, where offspring survival depends less on density‐dependent interactions and more on the chance that larvae are advected to a suitable early‐life environment, females may benefit from producing many relatively small eggs. In contrast, females in small systems with more consistent environments may benefit from producing relatively large eggs to increase early life provisions.

Despite hypotheses about how system size and associated environmental conditions should result in differences in egg size, few studies have examined these dynamics on an intraspecific basis, as fishes generally only spawn in either freshwater or saltwater. In many ways, very large freshwater lakes are analogous to marine systems (Pritt, Roseman, and O'Brien 2014). Consequently, early life‐stage fishes in large freshwater lakes may be exposed to environmental variability similar to marine systems. Like marine systems, very large freshwater lakes, such as the Laurentian Great Lakes, are often deep (e.g., Lake Michigan maximum depth is over 250 m), relatively oligotrophic, and have patchy species densities with greater diversity nearshore. One paradigm of marine recruitment dynamics is that marine species' egg and larval stages are adapted for dispersal (Leggett and Frank 2008). In large freshwater lakes, broad physical processes (e.g., upwellings, cross‐system circulation) may similarly affect larval dispersal. Local physical forces and broadscale currents can advect freshwater fish larvae considerable distances, causing them to settle and develop in locations up to 100 km from where spawning occurs (Dettmers et al. 2005; Höök et al. 2006; Beletsky et al. 2007; Oyadomari and Auer 2008; Zhao et al. 2009). Ludsin, DeVanna, and Smith (2014) suggested a need for further research on relationships between the physical environments of large freshwater ecosystems and the biology of their fish assemblages to make connections with the breadth of past marine research.

The similarities between large freshwater lakes and marine systems may select for similar trade‐offs between egg size and fecundity, and lead to production of relatively small eggs. However, unlike marine systems, large freshwater lakes may contain species that are present in smaller inland lakes as well. For example, in the Laurentian Great Lakes Region similar species are found across lakes and embayments of vastly different systems sizes, depths and productivities. This facilitates intraspecific comparisons of egg size across populations experiencing divergent environmental conditions, and evaluation of the expectation of optimal egg size theory (Smith and Fretwell 1974) and offspring size divergence proposed by Houde (1994). However, there is a paucity of such comparisons in published literature, likely because intraspecific comparisons of egg size across system sizes requires simultaneous sampling in multiple locations. There are numerous examples of egg size variation among fish populations (e.g., Morrongiello et al. 2012; Quinn, Hendry, and Wetzel 1995; Wang et al. 2012); however, these are typically comparisons among populations in similar system types and sizes.

While optimal egg size theory implies that the mean population egg size is adaptive, studies have suggested genetic divergences does not fully explain egg size variation (e.g., Katoh and Nishida 1994; Smoker et al. 2000), and egg size can exhibit substantial plasticity in response to environmental variation and maternal characteristics (Feiner, Wang et al. 2016). Intraspecific egg size may vary with factors such as temperature (Johnston and Leggett 2002; Jonsson and Jonsson 2016; Barneche, Burgess, and Marshall 2018), latitude (Fleming and Gross 1990; Beacham and Murray 1993; Kokita 2003), and productivity (Johnston and Leggett 2002; Wang et al. 2012), all of which vary among small and large freshwater systems. Annual variation in environmental conditions can influence mean population egg size across years (Tanasichuk and Ware 1987; Feiner, Wang et al. 2016), and controlled studies demonstrate that exposure to different temperature (Feiner, Malinich, and Höök 2018), food availability (Hutchings 1991), and competitive interactions can affect realized egg sizes. In addition, egg size varies within populations, often increasing in older, larger females (Duarte and Alcaraz 1989; Chambers and Leggett 1996; Hixon, Johnson, and Sogard 2014).

In this study, we compared average egg sizes of yellow perch (*Perca flavescens*) among populations in the Laurentian Great Lakes Region. Past studies demonstrate that walleye (*Sander vitreus*), another percid species, exhibit egg size variation spatially, among populations (Johnston and Leggett 2002; Wang et al. 2012) and temporally, within populations (Feiner, Wang et al. 2016), but such observations have not been reported for wild yellow perch populations, nor has egg size been compared across a broad gradient of system size. Like walleye (Johnston and Leggett 2002; Wang et al. 2012; Feiner, Wang et al. 2016), yellow perch display egg size variation within populations, through maternal effects, where egg size increases with female size (Lauer et al. 2005; Andree et al. 2015), according to female identity (Feiner, Malinich, and Höök 2018), and with variation in annual conditions, where females produce smaller eggs after shorter winter seasons (Farmer et al. 2015).

As our primary hypothesis, we predicted that yellow perch egg size would differ among populations and would vary negatively with system size. Further, we expected to observe variation of yellow perch egg size with female total length and potentially across years sampled. Specifically, we expected that yellow perch in large lakes (e.g., lakes Erie and Michigan) would exhibit small egg sizes relative to those from small lakes, similar to marine fishes described by Houde (1994), as they often experience extensive passive offshore transport in early life (Dettmers et al. 2005; Beletsky et al. 2007). In contrast, we predicted that populations inhabiting smaller inland lakes would produce relatively larger eggs, and yellow perch from medium‐sized systems would produce eggs of intermediate size.

## 2 Methods

### 2.1 Study Species

Yellow perch are native and ubiquitous throughout the Laurentian Great Lakes Region. Yellow perch are iteroparous, capital spawners (Malison et al. 1994; Henderson, Trivedi, and Collins 2000; Feiner and Höök 2015) and are characterized as displaying a periodic life history strategy (Winemiller and Rose 1992). Spawning occurs in the spring, but gonad development by mature females occurs essentially throughout the year and gonad size is typically determined by January (Henderson, Trivedi, and Collins 2000; Feiner and Höök 2015). Yellow perch produce a gelatinous skein that houses their eggs, and as demersal spawners, they drape their skeins across vegetation, woody debris, and large cobble substrates (Robillard and Marsden 2001; Feiner and Höök 2015). Mark‐recapture sampling efforts in Lake Michigan suggest that yellow perch demonstrate spawning site fidelity (Glover et al. 2008).

### 2.2 Sampling Sites

**FIGURE 1:**
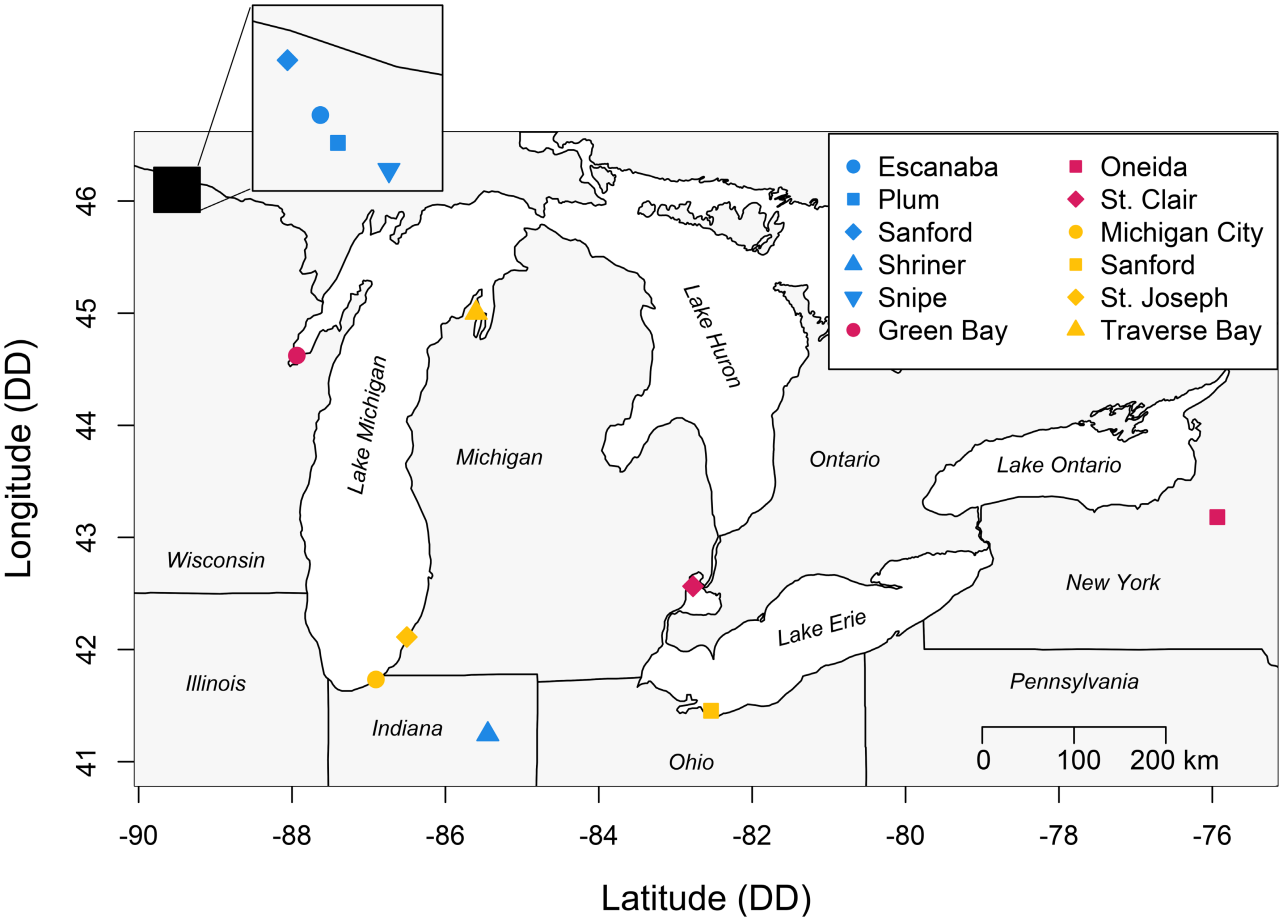
Map of sampling locations included in this study. Small (surface area < 1000 ha) systems are represented by blue points, medium (1000 < surface area < 500,000 ha) systems are represented by red points, and large (surface area > 500,000 ha) systems are represented by yellow points.

In spring (April—June, 2018, 2019 and 2023), we captured yellow perch during or just prior to spawning from 12 locations throughout the Great Lakes Region (Figure 1). The largest systems sampled were the main basins of Lake Erie (2,588,638 ha) and Lake Michigan (5,390,492 ha). We considered studies of Great Lakes yellow perch stock genetics to determine whether multiple locations could be considered as separate populations in our analyses. Larval yellow perch in these systems are subject to broadscale dispersal that allows for genetic admixture (e.g., Sepulveda‐Villet and Stepien 2011; Schraidt et al. 2023). In addition, we collected yellow perch from two embayments of Lake Michigan: Green Bay and Traverse Bay. Based on recent genomic evidence of population structure in Lake Michigan, we chose to consider Traverse Bay part of the main basin of Lake Michigan for surface area measurement and Green Bay as a separate system (Miller 2003; Schraidt et al. 2023). We sampled two lakes of intermediate size (1000 < Surface Area < 500,000 ha): Oneida Lake, New York, and Lake St. Clair, Michigan/Ontario. We sampled five lakes we classified as small (surface area < 1000 ha). Of the five inland lake locations, four were in northern Wisconsin (Escanaba Lake, Plum Lake, Sanford Lake, Snipe Lake, Vilas County), while the other was located in northeastern Indiana (Shriner Lake, Whitley County). The five small inland lakes ranged in size (37 to 432 ha; Table 1), with maximum depths ranging from 5 to 22 m. We measured the surface area of all locations using the polygon measurement tool in ArcGIS Online (Esri 2024; Appendix S1).

**TABLE 1:** Yellow perch sampling summary.

Location	Area (ha)	Year	*n*	TL (mm)	ED (mm)
Sanford Lake	37	2019	4	180 (150, 231)	1.347 (1.237, 1.534)
Shriner Lake	47	2018	20	300 (170, 344)	1.221 (1.008, 1.327)
—	—	2019	22	265 (196, 325)	1.276 (1.161, 1.390)
Snipe Lake	87	2019	10	171 (163, 186)	1.235 (1.161, 1.334)
Escanaba Lake	127	2019	40	236 (142, 320)	1.278 (1.130, 1.458)
—	—	2023	50	196 (140, 295)	1.272 (1.108, 1.377)
Plum Lake	432	2019	111	187 (142, 309)	1.169 (1.023, 1.455)
Oneida Lake	20,662	2023	40	289 (207, 344)	1.234 (1.063, 1.368)
Lake St. Clair	113,753	2023	7	260 (227, 306)	1.400 (1.328, 1.431)
Green Bay	420,425	2019	14	221 (146, 311)	1.200 (1.043, 1.306)
Sandusky	2,588,638	2023	60	285 (195, 350)	1.218 (1.006, 1.337)
St. Joseph	5,390,492	2018	4	220 (210, 225)	0.868 (0.850, 0.897)
Michigan City	5,390,492	2018	31	250 (133, 336)	0.915 (0.690, 1.178)
—	—	2019	21	266 (194, 358)	1.074 (0.987, 1.142)
—	—	2023	22	291 (226, 340)	1.145 (1.067, 1.247)
Traverse Bay	5,390,492	2018	6	205 (190, 221)	0.951 (0.874, 1.054)

*Note:* Each row represents a single collection year. *N* represents the number of observations of a given location and year. Sandusky is located in Lake Erie, and St. Joseph and Michigan City are located in Lake Michigan. Shriner Lake, Escanaba Lake, and Michigan City were sampled multiple years, indicated by dashes. Lake area was estimated using the polygon tool in ArcGIS. Total length (TL) and egg diameter (ED) are represented by means and corresponding (minimum, maximum).

### 2.3 Field Collection and Laboratory Processing

We collected spawning females via directed sampling led by Purdue University and in conjunction with monitoring programs led by state and federal natural resource agencies and Cornell University. To capture yellow perch, we used fyke nets, trap nets, bottom trawls, and benthic multifilament gill nets. This adaptive sampling approach using different methods was appropriate, as our aim was to collect sufficient numbers of spawning females and not to compare catch rates among locations. Sampling was conducted in accordance with all applicable laws set forth by governments and institutions, and all necessary scientific collector permits were acquired prior to sampling. For specific details related to capture methods used at each location, see Appendix S1. Upon capture, we measured total lengths of females to the nearest 1 mm. We collected approximately 10 mL of unfertilized eggs from each ripe female and immediately placed these samples in separate 20 mL scintillation vials with 10% neutral buffer formalin (i.e., we did not allow eggs to water harden).

We stored preserved egg samples for at least two months to standardize shrinkage (Feiner, Wang et al. 2016). After a two‐month period, we photographed a random subsample of 50–100 eggs from each female together with a standardized length scale (mm) under a dissecting microscope. Using ImageJ software, we measured vertical and horizontal diameters of 10 randomly selected eggs of each 50–100 subsample per female to the nearest 0.001 mm (Schneider, Rasband, and Eliceiri 2012; Andree et al. 2015; Feiner, Wang et al. 2016). We estimated an egg's overall diameter as the mean of vertical and horizontal diameters and calculated the average egg diameter for each female from these estimates. As such, the mean egg size for each female represents a single sample for subsequent statistical analyses.

### 2.4 Statistical Analyses

All statistical analyses were conducted using R statistical software version 4.1.2 (R Core Team 2021). For all tests, we determined significance at *α* = 0.05. Linear relationships between female size and egg size in fishes have been widely reported in published literature (Barneche et al. 2018; Koenigbauer and Höök 2023). Therefore, the size of females captured at each location could confound comparisons of mean egg size and should be accounted for. We tested the correlations between female total length and egg diameter with separate simple linear regressions for each sampling location where 10 or more females were collected. Additionally, we transformed these lake‐specific correlations to Fisher's *Z* effect sizes to compare with general patterns in freshwater fishes (Koenigbauer and Höök 2023). Further, Farmer et al. (2015) reported that annual effects (e.g., interannual environmental variation) can influence egg sizes of yellow perch across years. Because three sampling locations, Escanaba Lake, Michigan City (main basin of Lake Michigan), and Shriner Lake, were sampled in multiple years, we suspected such annual effects could contribute to variation in our egg diameter observations.

To compare yellow perch egg size along a gradient of system sizes, we used linear mixed models that accounted for maternal effects with a total length covariate and interannual variation as a random effect. Specifically, in each of our models we incorporated random intercepts for year. First, we compared mean egg diameter among lakes by including a categorical lake factor:

$$\mathrm{Egg}\;\text{diameter}\sim \text{Lake}\times \text{Total length}+\left(1|\text{Year}\right)$$

By including an interaction between lake and total length, we accounted for potential variation in maternal effects slopes among populations. The model also included lake and total length as separate predictor variables outside of the interaction. For this analysis, we only included observations from locations at which we collected at least ten samples (i.e., at least ten spawning females). We estimated mean egg diameters with 95% confidence intervals, then made pairwise comparisons of those means among lakes with the “emmeans” package (Lenth 2022). We estimated egg diameter ~ total length slopes with 95% confidence intervals for each lake using the “lstrends” function from the “emmeans” package. We organized pairwise comparisons of estimated marginal means and slopes with compound letter displays using the “cld” function of the “MuMIn” package (Barton and Barton 2015).

After our comparisons of mean egg diameter among lakes as a categorical factor, we more directly tested the pattern of decreasing egg size with increasing system size by replacing the lake factor with a continuous lake size variable, log_10_(lake surface area). Again, we accounted for maternal effects with a total length covariate and included a random year effect:

$$\mathrm{Egg}\;\text{diameter}\sim \mathrm{Log}\left(\text{Lake surface area}\right)\times \text{Total length}+\left(1|\text{Year}\right)$$

For this analysis, we incorporated all observations, including individuals from locations at which fewer than 10 samples were collected. All linear mixed models were run using the “afex” package (Singmann et al. 2015). To estimate marginal and conditional *R* ^2^ values for each mixed model, we used the “multcomp” package (Hothorn et al. 2016).

## 3 Results

**FIGURE 2:**
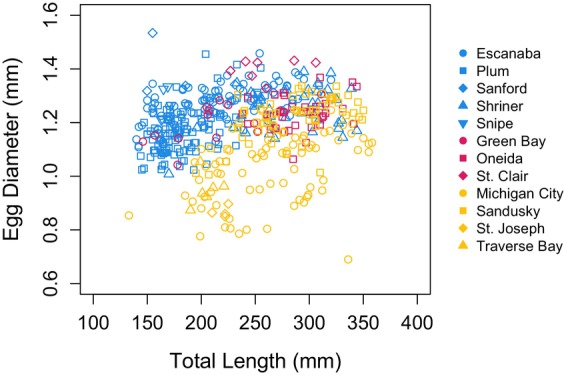
Scatterplot of individual mean yellow perch egg diameter measurements according to female total length from all locations and all years of our study. Small (surface area < 1000 ha) systems are represented by blue points, intermediate (1000 < surface area < 500,000 ha) systems are represented by red points, and large (surface area > 500,000 ha) systems are represented by yellow points.

During spring 2018, 2019, and 2023, we collected egg samples from 460 mature female yellow perch across 12 study locations (Table 1). Female total length ranged from 133 mm to 358 mm, and mean egg diameter per female ranged from 0.69 mm to 1.53 mm (Figure 2). We retained 439 observations from eight locations over three years for our model that compared mean egg diameter among lakes and used all 460 observations from 12 locations for our model that treated lake surface area as a continuous factor. In five of the eight locations where at least 10 females were collected, we found a significant, positive relationship between female total length and egg diameter (Table 2). Based on these tests, we observed a mean effect size (Fisher's *Z* = 0.426 ± SD 0.270) that was equal to the mean female size—egg diameter effect estimated across freshwater species (0.426), but was greater than the 95% confidence interval of a mean effect across other percid studies (0.167; Koenigbauer and Höök 2023).

**TABLE 2:** Summary statistics for simple linear regressions between female total length and egg diameter for locations with > 10 females sampled in our study.

Location	*n*	Slope	Intercept	*F* (df)	*p*	*R* ^2^	Fisher's *Z*
Shriner Lake	42	0.0006	1.0895	3.64 (1,40)	0.064	0.061	0.252
Snipe Lake	10	0.0000	1.2440	0.00 (1,8)	0.986	0.000	0.000
Escanaba Lake	88	0.0008	1.1039	38.89 (1,86)	< 0.001	0.303	0.619
Plum Lake	111	0.0017	0.8558	55.58 (1109)	< 0.001	0.332	0.657
Oneida Lake	40	0.0004	1.1193	1.54 (1,38)	0.222	0.014	0.119
Green Bay	14	0.0010	0.9799	8.69 (1,12)	0.012	0.372	0.709
Sandusky	60	0.0011	0.8994	25.53 (1,58)	< 0.001	0.294	0.607
Michigan city	74	0.7344	0.0011	16.47 (1,72)	< 0.001	0.175	0.446

**FIGURE 3:**
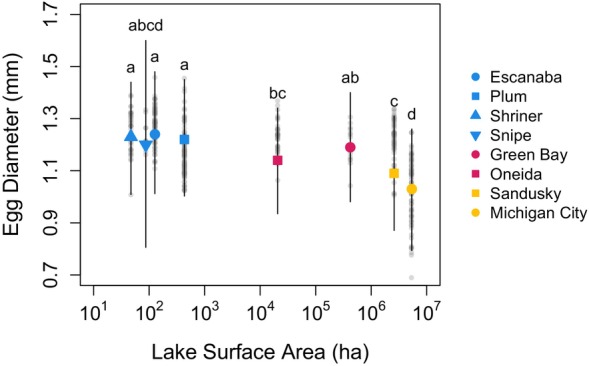
Estimated mean egg diameter and 95% confidence intervals by lake from a linear mixed model including lake as a categorical factor, total length as a covariate, and a random year effect. Degrees of freedom for estimated marginal means were calculated using the Kenward‐Roger method. Only lakes with *n* > 10 observations were included in this analysis. Each lake mean and confidence interval were plotted according to their respective log (surface area). Small (surface area < 1000 ha) systems are represented by blue points, medium (1000 < surface area < 500,000 ha) systems are represented by red points, and large (surface area > 500,000 ha) systems are represented by yellow points. Lakes with different letters represent significant pairwise differences in mean egg diameter (*p* < 0.05). Smaller gray points in the background represent individual egg diameter observations for females from each lake.

Our linear mixed model that included a categorical lake factor explained 78.3% of variation of egg diameter in our dataset ($R_{\text{Conditional}}^{2}$), with 33.0% explained by just fixed effects ($R_{\text{Marginal}}^{2}$). Lake (*F*_7,421_ = 2.04, *p* = 0.049), total length (*F*_1,421_ = 5.85, *p* = 0.016), and their interaction (*F*_7,421_ = 3.19, *p* = 0.003) were all significant predictors of egg diameter. Random intercepts for yellow perch collected in 2018, 2019, and 2023 were − 0.105, 0.029, and 0.077, respectively. Estimated mean egg diameters from each lake varied (Table S1) and characterized a pattern of egg diameter decreasing in larger systems (*p* < 0.05; Figure 3). As indicated by the significant interaction term, slopes of the egg diameter total length relationship varied among lakes, but such slope differences were apparent in only two of 28 pairwise comparisons (Figure S1). In those two pairwise comparisons, the egg diameter ~ total length slope was greater in Plum Lake than in Michigan City and Oneida Lake (*p* < 0.05; Figure S1). If we removed Plum Lake observations from the analysis, including all locations, the interaction term became insignificant, but the lake and total length factors remained significant.

**FIGURE 4 |:**
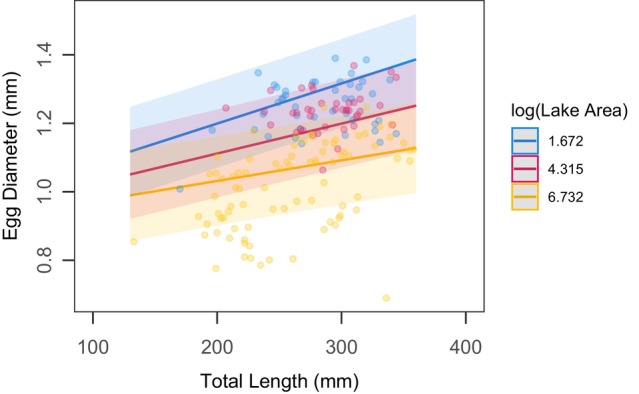
Estimated egg diameter vs. total length trendlines and 95% confidence intervals from a linear mixed model including log (lake surface area) as a continuous factor, total length as a covariate, and a random year effect. Observations from all lakes sampled, including those with *n* < 10 observations were included in this analysis. We plotted representative slopes from small (blue; Shriner Lake), medium (red; Oneida Lake), and large (yellow; Lake Michigan) lakes. Points represent individual female observations from each lake.

Our linear mixed model that included a continuous lake surface area factor explained 75.5% of variation of egg diameter in our dataset ($R_{\text{Conditional}}^{2}$), with 23.0% explained by just fixed effects ($R_{\text{Marginal}}^{2}$). Log_10_(lake surface area) did not significantly predict egg diameter (*F*_1,455_ 1.07, *p* = 0.301); however, total length (*F*_1,455_ = 68.31, *p* < 0.001), and the interaction of lake surface area and individual female length (*F*_1,455_ = 8.11, *p* = 0.005) were significant. Random intercepts for yellow perch collected in 2018, 2019, and 2023 were −0.125, 0.031, and 0.093, respectively. To interpret the significant interaction, we plotted egg diameter ~ total length slopes at different values of log_10_ (lake surface area) (Figure 4). We found that slopes were greater in lakes with a smaller surface area, suggesting that egg diameter did not vary among lakes for females with shorter total length, but did vary among lakes when considering larger females with greater lengths (Table S1).

## 4 Discussion

Through our examination of egg diameter variation in a single species across a gradient of system size spanning four orders of magnitude in surface area, we found a pattern of differential maternal investment in individual offspring that is consistent with past hypotheses related to interspecific comparisons between marine and freshwater systems. The difference in egg diameter (accounting for maternal total length effects) among populations inhabiting relatively small and large systems was substantial. In our general linear model that compared yellow perch egg diameter among lakes as a categorical factor, the largest estimated mean diameter (Escanaba Lake, 1.24 mm) was 20.4% larger than the smallest estimated mean diameter (Michigan City, 1.03 mm). Assuming yellow perch eggs are perfectly spherical and transforming egg diameter to egg volume, the magnitude of difference becomes even larger with Escanaba Lake eggs (1.00 mm^3^) being 74.5% larger than Michigan City (0.572 mm^3^). Considering the range of average egg diameter per female in our study (0.69–1.534 mm), there is potential for yellow perch egg volume to vary at an even greater magnitude. Such differences in egg size imply varying strategies for the trade‐off between egg size and fecundity (Einum and Fleming 2000; Kamler 2005).

This pattern of egg size varying with system size was generally consistent with comparisons between freshwater and marine fishes but required more careful interpretation. The significant interaction term in our model that considered lake surface area as a continuous factor indicated that maternal length effects on egg diameter (slopes) were weaker as lake surface area increased. This pattern could reflect potential differences in reproductive strategies, where Sweepstakes Reproductive Success (Hedgecock and Pudovkin 2011) favors increased fecundity over individual provisioning in systems with highly variable larval environments (i.e., Great Lakes and marine systems). Consideration of this interaction suggested that egg diameter variation among different‐sized lakes was less apparent in shorter females but was realized once females became longer. Among longer females, individuals from relatively large lakes produced smaller eggs than individuals from relatively small lakes. This may further reflect variation in reproductive strategy, where the longest females with the most energy to invest in gonadal development prioritize fecundity over individual provisioning when offspring survival is more stochastic. Overall, these results supported our expectation that intraspecific differences in egg size across a gradient of system sizes would be consistent with interspecific patterns of egg size (Duarte and Alcaraz 1989) and larval size (Houde 1994) observed between freshwater and marine fishes.

Our findings suggest that the physical and ecological forces driving early‐life performance and offspring size variation of fishes in marine versus freshwater systems may also contribute to variation in offspring size among freshwater systems of different sizes. Different species of fish may have widely varying evolutionary histories, spawning behaviors, and life history strategies, which could affect observed interspecific differences in egg size. For example, comparisons across freshwater and marine species may include fish with either pelagic or demersal spawning strategies, which may play a role in egg size patterns (Lønning, Køjrsvik, and Falk‐petersen 1988). By comparing egg size across system sizes within a species, we reduced potential factors affecting past interspecific studies. These past studies characterized marine fishes' relatively small eggs and larvae as an adaptation related to broadscale offspring transport in early life (Leggett and Frank 2008). We found that yellow perch in Great Lakes, especially Lake Michigan, which are similarly subject to broadscale dispersal in early life (Höök et al. 2006; Beletsky et al. 2007; Schraidt et al. 2023), produce relatively small eggs like marine fishes, whereas yellow perch populations inhabiting smaller systems with lower dispersal potential produced larger eggs.

Offspring size has been hypothesized to vary with environment, and the trade‐off between fecundity and egg size likely reflects adaptation to local environments (Einum and Fleming 2002; Smith and Fretwell 1974). We did not explicitly evaluate genetic differences among populations and genetic influence on egg size. Feiner, Malinich, and Höök (2018) found that within a population held in a research pond, individual identity strongly influenced yellow perch egg size across years, which indicates potential for genetic contribution to egg size variation. To the best of our knowledge, we accounted for population structure in our analyses. Unlike several other species targeted by recreational fishing, yellow perch do not have an extensive stocking history in our study region. Lake Michigan yellow perch populations are generally genetically distinct from inland yellow perch (Sepulveda‐Villet and Stepien 2011). Further, yellow perch have been found to differentiate genetically between upper and lower Great Lakes (Sepulveda‐Villet and Stepien 2012; Sullivan and Stepien 2014). Moreover, previous work on the genetic structure of yellow perch has shown that main basin Lake Michigan and Green Bay populations are genetically distinct (Miller 2003; Sepulveda‐Villet et al. 2009; Schraidt et al. 2023) and exhibit different reproductive life history patterns (Feiner et al. 2015). While we suggest that the populations included in our analyses were genetically distinct, we could not attribute resulting patterns in egg diameter variation to just one of genetic divergence or plasticity to environmental conditions. We predict the genetically similar populations may have more similar adapted mean egg diameter. However, it is also possible that within‐population genetic diversity could inform egg size variation, where more diverse populations could express greater egg size variation or maternal effects. Perhaps this could explain why we found significant maternal effects in some but not all lakes sampled.

Our observed egg size patterns also align with temporal and spatial environmental variation. Glover et al. (2008) reported that adult yellow perch in Lake Michigan dispersed up to 100 km throughout the year. During such dispersal, it is possible that females experience variable environments during oogenesis, which may contribute to the development of smaller eggs. However, the environment females experience during oogenesis could be greatest in nearshore habitats, implying such effects could take place in individuals with less dispersal, too. Further, energy expenditures of dispersal could limit gonadal investment more in large systems than in small systems. Farmer et al. (2015) reported that yellow perch from Lake Erie produced different‐sized eggs according to the annual duration of winter, suggesting that after longer winters, female yellow perch produced larger eggs on average. Similarly, in a multi‐year study of a yellow perch population in a research pond, Feiner, Malinich, and Höök (2018) demonstrated an annual effect on egg size. Additionally, in aquaculture settings, Feiner, Coulter et al. (2016) found that within‐female egg size variation of yellow perch was greater when reared in warmer temperatures. In this study, we observed that the random effect of sampling year explained a considerable amount of variation in egg diameter. The average water surface temperature of Lake Michigan, which likely correlates with the average regional temperature throughout the Great Lakes Region, during the first 120 days of the year increased across years (2.179 C in 2018, 2.294 C in 2019, 3.201 C in 2023; NOAA 2024). For both general linear models, random intercepts indicated that yellow perch eggs were larger in 2018 than 2019 and 2023 (i.e., negative random intercepts adjusted 2018 egg diameter observations to be closer to warmer years), which aligns with Farmer et al. (2015).

Like other studies (e.g., Lauer et al. 2005; Andree et al. 2015), we detected a positive relationship between female length and egg diameter in yellow perch individually in five of eight lakes tested, and total length was a significant predictor of egg diameter in both of our general linear models. Maternal effects, or the non‐genetic relationship between the phenotype of mothers and their offspring, have been widely reported in fishes (Green 2008), including percid species similar to yellow perch, such as congeneric Eurasian perch (*Perca fluviatilis*, Olin et al. 2012), pikeperch (*Sander lucioperca*, Olin et al. 2021), and walleye (Feiner, Wang et al. 2016). In lakes where we found significant positive relationships between female total length and egg diameter, corresponding effect sizes (Fisher's *Z*) were similar to what was generally observed in freshwater species (Koenigbauer and Höök 2023). These maternal effects likely play a role in the reproductive potential of individual females and may be a contributing factor for differential early life performance (Smith and Fretwell 1974; Scott, Marteinsdottir, and Wright 1999; Hixon, Johnson, and Sogard 2014). Hixon, Johnson, and Sogard (2014) suggested that annual recruitment of populations that demonstrate strong maternal effect relationships benefits from the presence of larger, older females. Therefore, some authors have called for management of fisheries to focus on protection of particular age‐ and size‐class distributions (Berkeley et al. 2004; Gwinn et al. 2015). Similar considerations may be beneficial for yellow perch. Past studies have shown a positive relationship between egg size and offspring survival in percid species (Moodie et al. 1989; Venturelli et al. 2010; Koenigbauer and Höök 2023). Heyer et al. (2001) found that survival of larvae to the adult stage, and thus, recruitment, varied with maternal effects of yellow perch. However, Andree et al. (2015) reported that despite a positive relationship between female yellow perch size and egg size, there were negative relationships between maternal size and larval growth and survival.

Our observation of intraspecific egg size variation along a very broad gradient of system size is to our knowledge the first of its kind and extends upon past interspecific comparisons between freshwater and marine fishes. The pattern of small eggs in larger systems potentially reflects an optimal egg size trade‐off, where producing many small eggs is suitable in habitats where offspring experience highly variable environments due to broadscale larval dispersion, but producing fewer large eggs is suitable in smaller, more stable larval environments. Further, our finding of interpopulation egg size variation, along with observations of maternal effects in yellow perch, reflect similar studies but also expand our understanding of percids. Multiple examples exist of interpopulation variation of egg size in walleye (Johnston and Leggett 2002; Wang et al. 2012; Feiner, Wang et al. 2016; and Heibo, Magnhagen, and Vøllestad 2005) found variation among populations in reproductive investment of congeneric Eurasian perch. However, this study may be the first evidence of interpopulation variation of egg size in wild yellow perch. Our observed significant interactions in both general linear models suggested that egg size variation may be greater in longer females, which may reflect increased energetic reserves to develop larger eggs as females grow. Additionally, they suggest the strength of maternal effects may vary with system size. Continuing to assess fishes' local adaptation of reproductive traits to their physical environments, especially when a single species such as yellow perch inhabits vastly different‐sized systems, may inform our understanding of reproductive ecology and improve our ability to predict recruitment and effectively manage fisheries in the future.

## Author Contributions

**Scott T. Koenigbauer:** conceptualization (lead), data curation (lead), formal analysis (lead), investigation (lead), methodology (lead), visualization (lead), writing – original draft (lead), writing – review and editing (lead). **Zachary S. Feiner:** conceptualization (equal), formal analysis (equal), investigation (equal), methodology (equal), writing – original draft (equal), writing – review and editing (equal). **Benjamin Dickinson:** data curation (equal), investigation (equal), resources (equal), writing – original draft (equal), writing – review and editing (equal). **Stephanie L. Shaw:** data curation (equal), resources (equal), writing – original draft (equal), writing – review and editing (equal). **L. Zoe Almeida:** data curation (equal), investigation (equal), resources (equal), writing – review and editing (equal). **Mark R. DuFour:** data curation (equal), formal analysis (equal), investigation (equal), resources (equal), writing – original draft (equal), writing – review and editing (equal). **Alexander J. Gatch:** data curation (equal), investigation (equal), writing – original draft (equal), writing – review and editing (equal). **Claire Schraidt:** data curation (supporting), investigation (supporting), writing – review and editing (supporting). **Tomas O. Höök:** conceptualization (equal), formal analysis (equal), funding acquisition (lead), investigation (equal), methodology (equal), project administration (lead), supervision (lead), writing – original draft (equal), writing – review and editing (equal).

## Ethics Statement

All handling of live animals for this study was approved by the Purdue Institutional Animal Care and Use Committee (PACUC), and all necessary permits and approvals for sampling were obtained from each sampling locations' respective natural resource management agency.

## Conflicts of Interest

The authors declare no conflicts of interest.

## Supporting information


Appendix S1

## Data Availability

Fish sampling data are available with open access through the Purdue University Research Repository (https://doi.org/10.4231/A420‐R024). The R code for statistical analyses can be found in Appendix S1.
