# Supplementary material for: Egg Size Scales Negatively With System Size in a Periodic Fish Species
Source: Ecol Evol. 2024 Oct 17;14(10):e70426. doi: 10.1002/ece3.70426 (PMC11486665; doi:10.1002/ece3.70426)
Supplement: Supplementary file 1 — Appendix S1 [file ECE3-14-e70426-s001.docx]

**Supplemental Information**

**Lake Surface Area Measurements**

Lake Michigan (Michigan City and St. Joseph)


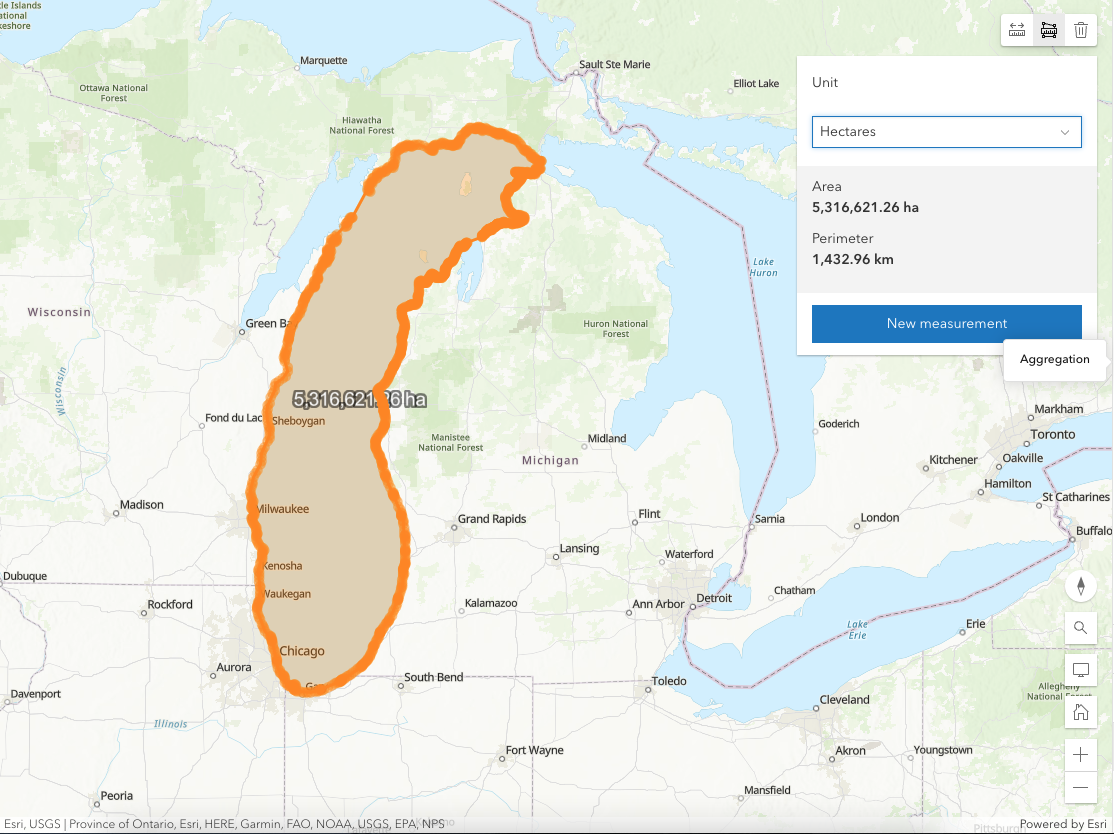


Lake Erie (Sandusky)


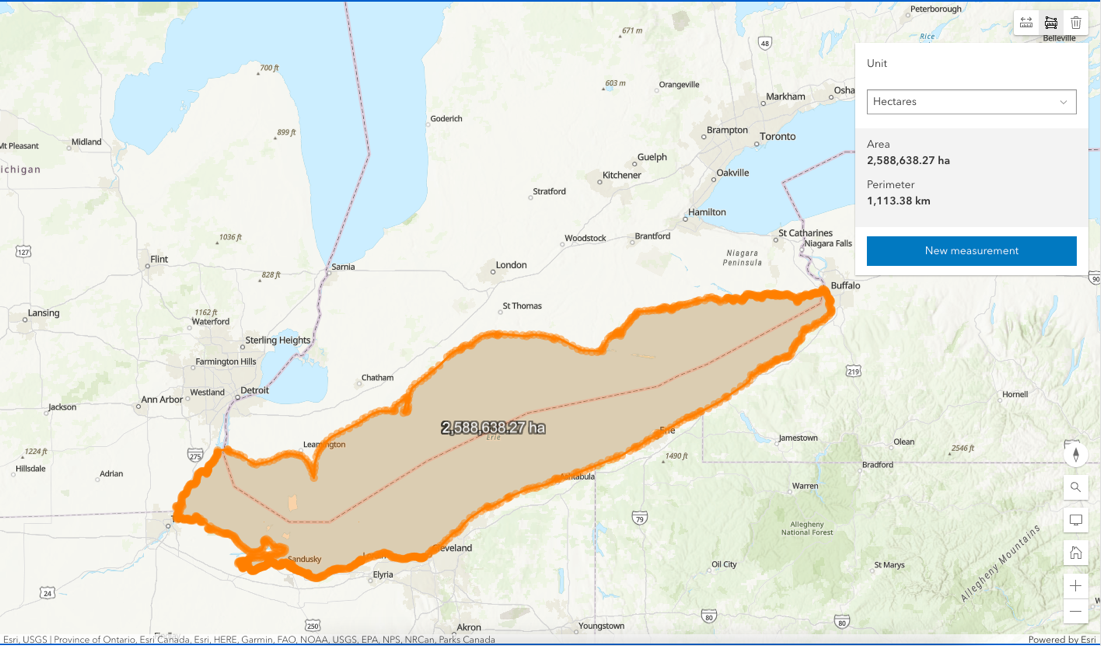


Green Bay


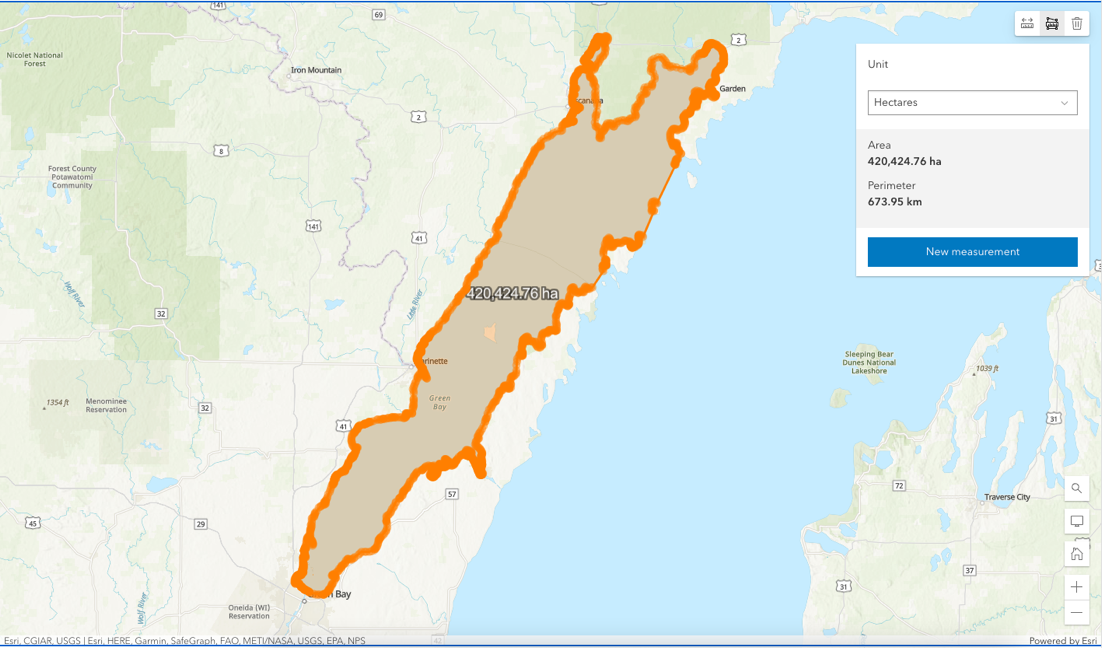


Lake St. Clair


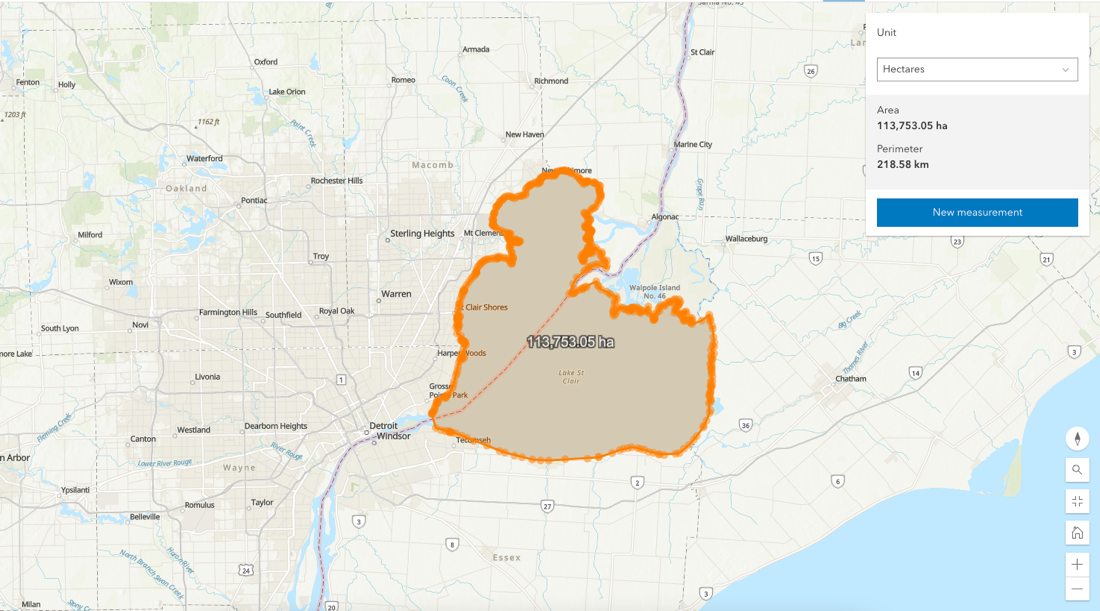


Traverse Bay (We combined the Traverse Bay and Lake Michigan measurements based on genetic similarity of yellow perch)


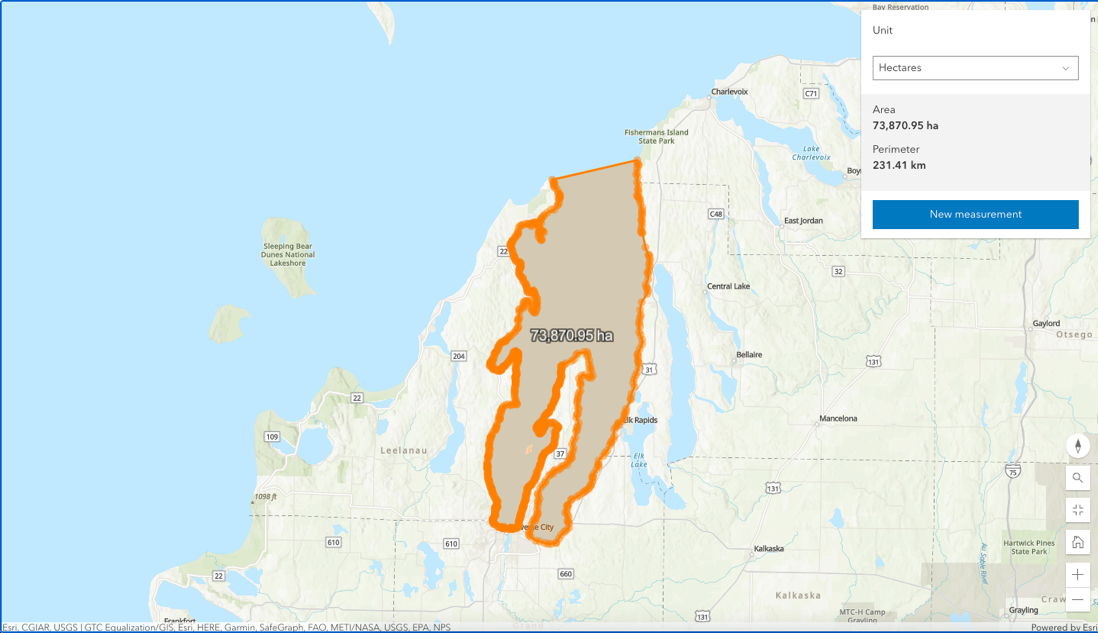


Oneida Lake


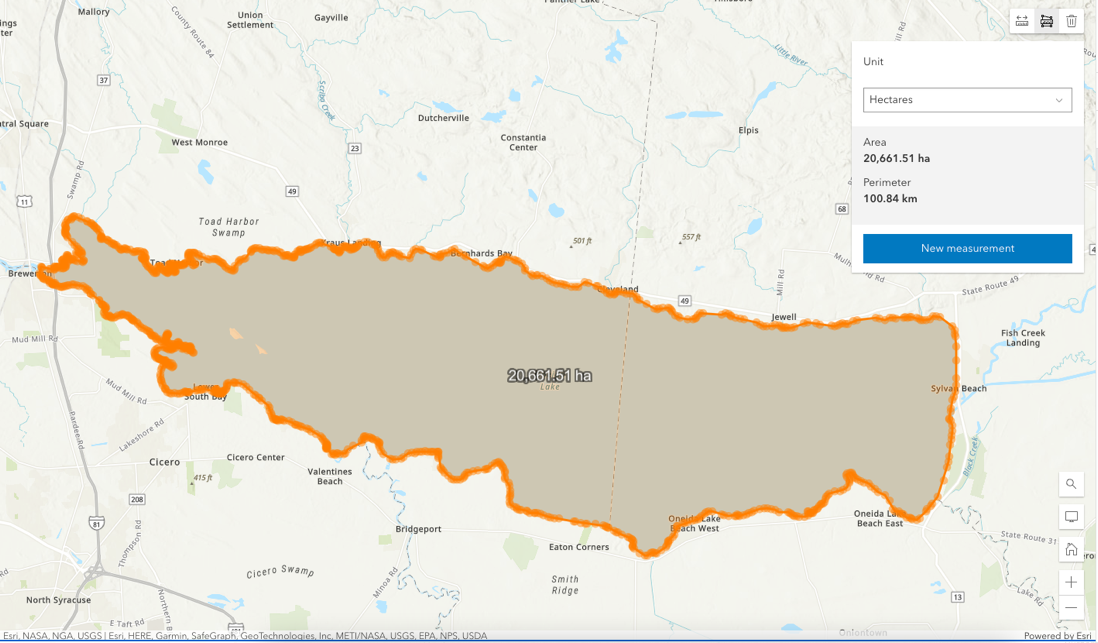


Plum Lake


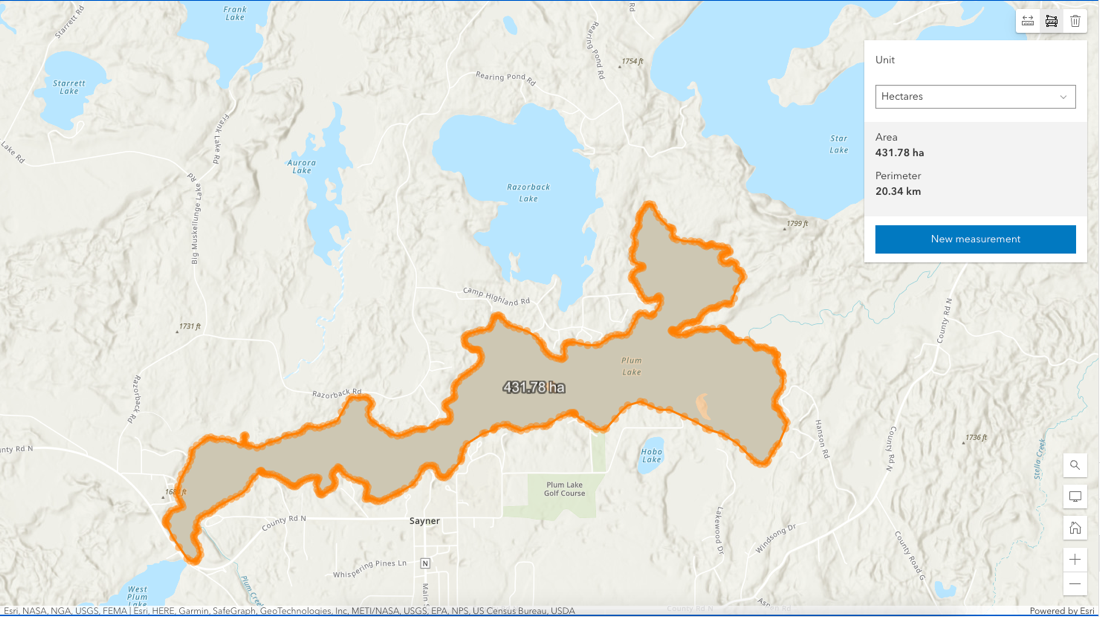


Escanaba Lake


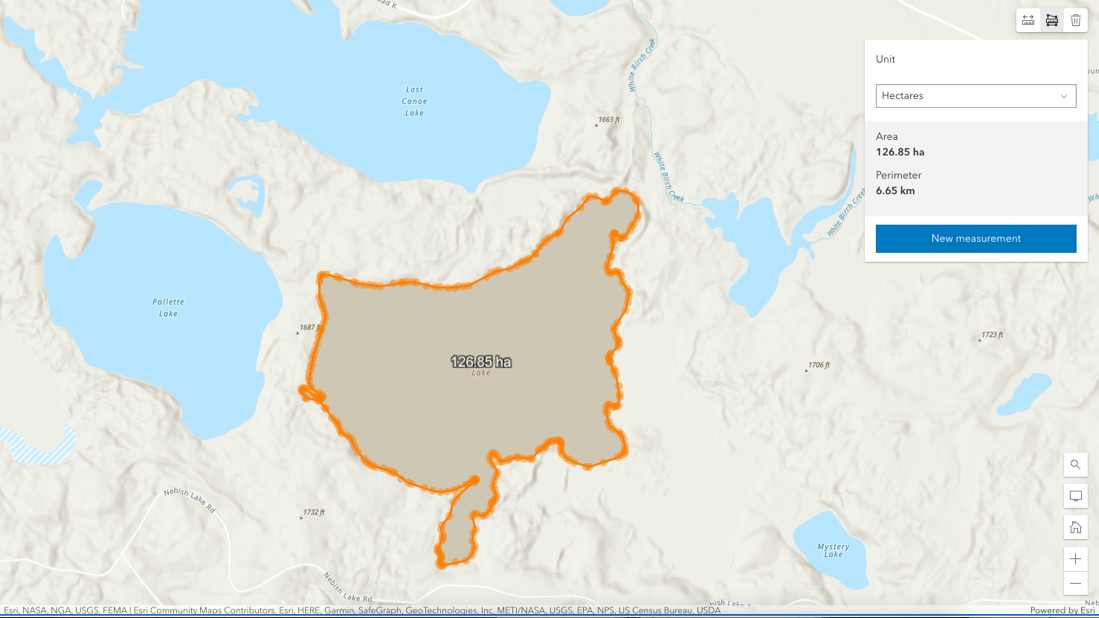


Snipe Lake


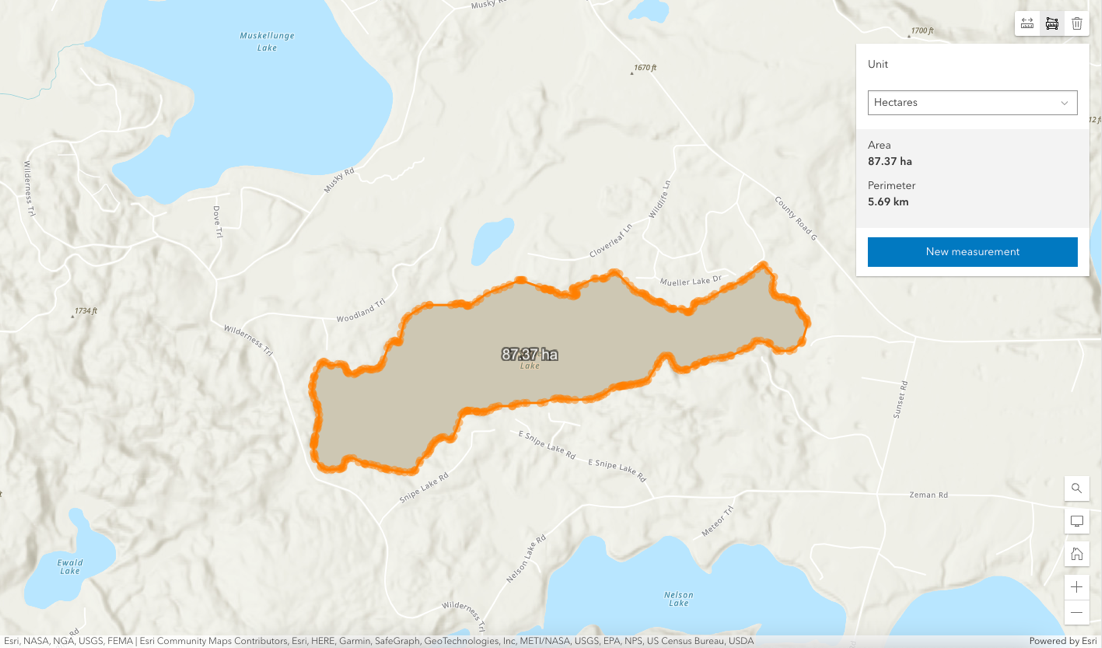


Shriner Lake


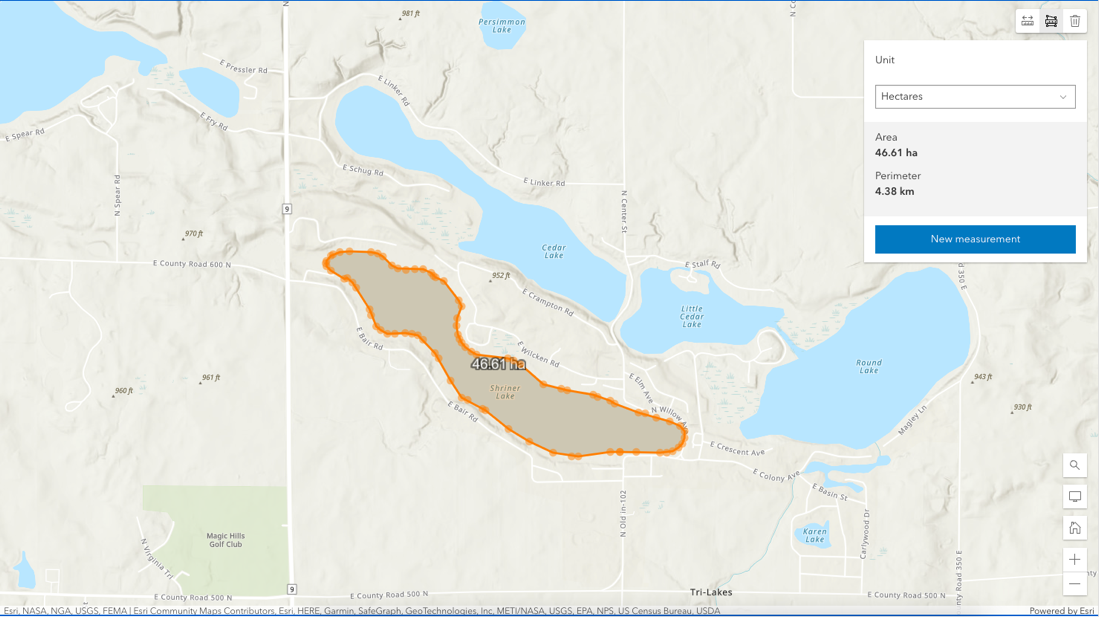


Sanford Lake


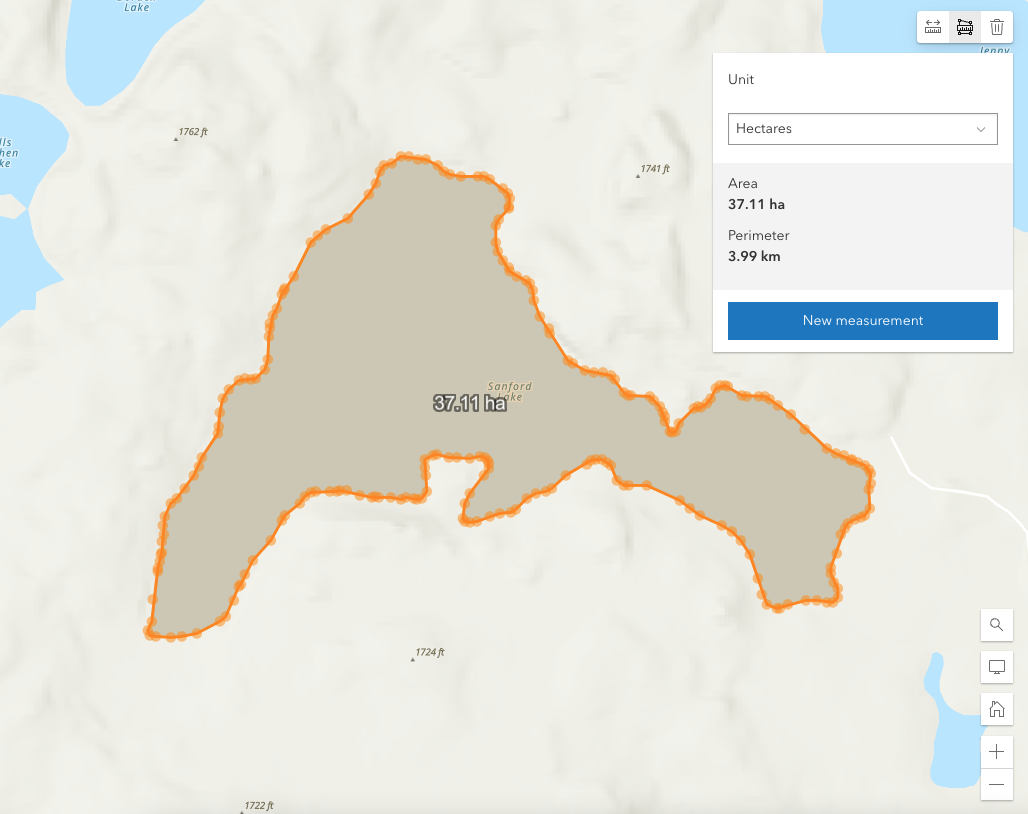


**Sampling gear used at each location**

*Wisconsin inland – Plum Lake, Escanaba Lake, Snipe Lake and Sanford Lake*

In inland waters of Wisconsin, Wisconsin Department of Natural Resources (WIDNR) deployed fyke nets (1.2 × 1.8 m frame; 19.1 mm mesh) immediately after ice-out for 4-14 days.

*Indiana inland – Shriner Lake*

Purdue University set overnight experimental benthic experimental gill nets with three mesh sizes (51, 64 and 76 mm). The gill nets had three panels of each mesh size in a repeating pattern. Nets were set perpendicular to shore from approximately 2- to 15-meter depths.

*Lake Michigan – Michigan City and St. Joseph*

In the main basin of Lake Michigan, Purdue University and Indiana Department of Natural Resources (INDNR) set overnight experimental benthic experimental gill nets with three mesh sizes (51, 64 and 76 mm). The gill nets had three panels of each mesh size in a repeating pattern. Gill nets were set parallel to shore at 10- and 15-m depth,

*Green Bay*

WIDNR set double-ended fyke nets at 2- to 3-m depth.

*Lake Erie – Sandusky Sub-basin*

U.S. Geological Survey was provided yellow perch samples captured on 4/21/2023 from an Ohio Department of Natural Resources bottom trawl (Yankee two-seam bottom trawl with a 10.4-

m head rope, 25-mm bar mesh in the cod end, 13-mm stretched mesh liner, and 25.4-cm roller gear) near the Lorain Ridge (- 41.58333, -82.33333). U.S. Geological survey was provided yellow perch captured by commercial trap net on 4/27/2023 and 5/10/2023 at nearshore (- 41.41764, -82.58382) and offshore (- 41.53917, -82.50378) locations, respectively. All samples were collected within Lake Erie’s Sandusky Sub-basin.

*New York inland – Oneida Lake*

In Oneida Lake, yellow perch were collected with two gears: Oneida trap nets and multifilament gillnets. The trap nets were set perpendicular to shore near Constantia, New York by the New York State Department of Environmental Conservation to capture walleye during spawning for use in the Oneida Fish Hatchery. The lead was inshore and the car was offshore at approximately 2.5 m depth. Net dimensions included the 1.83 × 1.83 × 1.83 m car, 12.8 m wings, and a 45.7 m lead. All sections were constructed of 25.4 mm bar mesh multifilament netting. Yellow perch were bycatch in this effort and only yellow perch collected on 12 April 2023 were used. The gill nets were set by the Cornell University Biological Field Station over two days (11-12 April 2023). Gill nets were set during the day for approximately 6-hours, dimensions were 45.7 × 1.8 m, and mesh sizes ranged from 89–152 mm.

*Lake St. Clair*

Michigan Department of Natural Resources (MIDNR) deploys trap nets that are 200 m long and 2 m tall in Anchor Bay, Lake St. Clair. Nets are deployed in depths greater than 3 m between April – May.

**Linear Mixed Model Pairwise Comparisons**

Supplemental Table 1. Summary statistics from pairwise Tukey Contrasts of yellow perch egg diameter among locations according to linear mixed model including total length covariate and random year effect. Negative estimate indicates that the first location listed has a smaller mean egg diameter than the second location listed.

| **Pair** | **Estimate** | **SE** | **t Ratio** | **p** |
| --- | --- | --- | --- | --- |
| Erie - Escanaba | -0.154 | 0.017 | -9.195 | < 0.001 |
| Sandusky - Green Bay | -0.099 | 0.026 | -3.795 | 0.004 |
| Sandusky - Michigan City | 0.064 | 0.019 | 3.410 | 0.016 |
| Sandusky - Oneida | -0.047 | 0.025 | -1.915 | 0.541 |
| Sandusky - Plum | -0.135 | 0.022 | -6.238 | < 0.001 |
| Sandusky - Shriner | -0.137 | 0.023 | -5.869 | < 0.001 |
| Sandusky - Snipe | -0.114 | 0.195 | -0.583 | 0.999 |
| Escanaba - Green Bay | 0.055 | 0.021 | 2.616 | 0.153 |
| Escanaba - Michigan City | 0.218 | 0.014 | 15.990 | < 0.001 |
| Escanaba - Oneida | 0.107 | 0.023 | 4.552 | < 0.001 |
| Escanaba - Plum | 0.019 | 0.016 | 1.228 | 0.923 |
| Escanaba - Shriner | 0.018 | 0.018 | 0.978 | 0.977 |
| Escanaba - Snipe | 0.041 | 0.194 | 0.209 | 1.000 |
| Green Bay - Michigan City | 0.163 | 0.022 | 7.314 | < 0.001 |
| Green Bay - Oneida | 0.051 | 0.031 | 1.670 | 0.707 |
| Green Bay - Plum | -0.036 | 0.022 | -1.625 | 0.735 |
| Green Bay - Shriner | -0.038 | 0.024 | -1.551 | 0.779 |
| Green Bay - Snipe | -0.015 | 0.195 | -0.076 | 1.000 |
| Michigan City - Oneida | -0.112 | 0.025 | -4.469 | < 0.001 |
| Michigan City - Plum | -0.199 | 0.017 | -11.738 | < 0.001 |
| Michigan City - Shriner | -0.201 | 0.018 | -10.980 | < 0.001 |
| Michigan City - Snipe | -0.178 | 0.194 | -0.915 | 0.985 |
| Oneida - Plum | -0.088 | 0.027 | -3.229 | 0.029 |
| Oneida - Shriner | -0.089 | 0.029 | -3.132 | 0.039 |
| Oneida - Snipe | -0.066 | 0.195 | -0.339 | 1.000 |
| Plum - Shriner | -0.001 | 0.020 | -0.073 | 1.000 |
| Plum - Snipe | 0.022 | 0.194 | 0.111 | 1.000 |
| Shriner - Snipe | 0.023 | 0.195 | 0.118 | 1.000 |

Supplemental Figure1. Estimated egg diameter vs. total length slopes and 95% confidence intervals by lake from mixed model 1. Only lakes with n > 10 observations were included in this analysis. Each lake mean and confidence interval were plotted according to their respective log(Surface Area). Small (Surface Area < 1,000 ha) systems are represented by blue points, medium (1,000 < Surface Area < 500,000 ha) systems are represented by red points, and large (Surface Area > 500,000 ha) are represented by yellow points. Lakes with different letters represent significant pairwise differences in slopes (p < 0.05).

Supplemental Table 2. Estimated yellow perch mean egg diameter (mm) and Egg Diameter ~ Total Length slopes for each lake included in the general linear model: Egg Diameter ~ log(Surface Area) * Total Length + (1|Year).

| **Lake** | **log_ha** | **Mean ED (mm)** | **Slope** | **SE** | **df** | **lower.CL** | **upper.CL** |
| --- | --- | --- | --- | --- | --- | --- | --- |
| Sanford | 1.568 | 1.250 | 0.00118 | 0.00011 | 454.76 | 0.00096 | 0.0014 |
| Shriner | 1.672 | 1.240 | 0.00117 | 0.00011 | 454.75 | 0.00096 | 0.0014 |
| Snipe | 1.940 | 1.230 | 0.00114 | 0.00010 | 454.71 | 0.00094 | 0.0013 |
| Escanaba | 2.095 | 1.230 | 0.00112 | 0.00010 | 454.68 | 0.00093 | 0.0013 |
| Plum | 2.643 | 1.210 | 0.00106 | 0.00008 | 454.54 | 0.00090 | 0.0012 |
| Oneida | 4.318 | 1.140 | 0.00087 | 0.00008 | 454.26 | 0.00072 | 0.0010 |
| St. Clair | 5.057 | 1.120 | 0.00079 | 0.00009 | 454.39 | 0.00061 | 0.0010 |
| Green Bay | 5.620 | 1.100 | 0.00073 | 0.00010 | 454.51 | 0.00052 | 0.0009 |
| Erie | 6.411 | 1.070 | 0.00064 | 0.00013 | 454.63 | 0.00039 | 0.0009 |
| Michigan | 6.732 | 1.050 | 0.00060 | 0.00014 | 454.66 | 0.00033 | 0.0009 |

**R Code for Statistical Models**

######R script for "Egg size scales negatively with system size in a periodic fish species", Koenigbauer et al. 2024

yp1<-read.csv("~/YP_Eggs.csv") #adjust path to read in .csv data file

yp1$Year<-as.factor(yp1$Year) #Ensure year is treated as factor

yp<-yp1[c(1:185,196:266,274:459),] #Omit lakes where n < 10 for analyses 1 and 2

###Analysis 1: Examine maternal effects relationships in lakes where n > 10

#Create data subsets for lakes of interest

shr<-yp1[which(yp1$Lake=='Shriner'),]

snp<-yp1[which(yp1$Lake=='Snipe'),]

esc<-yp1[which(yp1$Lake=='Escanaba'),]

plm<-yp1[which(yp1$Lake=='Plum'),]

one<-yp1[which(yp1$Lake=='Oneida'),]

gb<-yp1[which(yp1$Lake=='Green Bay'),]

eri<-yp1[which(yp1$Lake=='Erie'),]

mic<-yp1[which(yp1$Lake=='Michigan City'),]

#Fit simple linear regressions for linear relationships between Egg Diameter and Total length, along with correlations

lmshr<-lm(shr$EDmm~shr$TLmm)

lmsnp<-lm(snp$EDmm~snp$TLmm)

lmesc<-lm(esc$EDmm~esc$TLmm)

lmplm<-lm(plm$EDmm~plm$TLmm)

lmone<-lm(one$EDmm~one$TLmm)

lmgb<-lm(gb$EDmm~gb$TLmm)

lmeri<-lm(eri$EDmm~eri$TLmm)

lmmic<-lm(mic$EDmm~mic$TLmm)

summary(lmshr) #Use the summary() function to extract regression coefficients, Rsq

###Analysis 2: Linear mixed model to compare mean egg size among lakes, lake treated as a categorical factor

#Packages for linear mixed model

library(afex)

library(MuMIn)

#Model 1, include Year as a random factor, note used "yp" and not "yp1" to exclude lakes with n < 10 yellow perch

m1<-mixed(EDmm~TLmm*Lake+(1|Year),data=yp,method="KR",control=lmerControl(optCtrl = list(maxfun= 1e6)),expand_re=TRUE)

anova(m1)

r.squaredGLMM(m1$full_model) #Marginal and conditional Rsq for % variance explained by model

m1lmer<-lmer(EDmm~TLmm*Lake+(1|Year),data=yp) #use lmer() instead of mixed() for random year intercepts

ranef(m1lmer) #extract random year intercepts

#Packages for pairwise comparisons of marginal means

library('emmeans')

library('multcomp')

library('multcompView')

emm_options(lmer.df="kenward-roger") #Adjust comparison method to Kenward-Roger

m1_emm<-emmeans(m1,"Lake",model="multivariate",adjust="bonferroni") #Bonferroni correction of p-values

pairs(m1_emm) #Pairwise comparison of lake marginal means

cld(m1_emm) #Organize pairs by significance

m1_slopes<-lstrends(m1,"Lake",var="TLmm",adjust="tukey") #Extract slopes with 95% confidence intervals for each lake

###Analysis 3: Linear mixed model to compare mean egg size along lake surface area as a continuous factor

#Model 2, include Year as a random factor, note used "yp1" and not "yp" to include lakes with n < 10 yellow perch

m2<-mixed(EDmm~TLmm*log_ha+(1|Year),data=yp1,method="KR",control=lmerControl(optCtrl = list(maxfun= 1e6)),expand_re=TRUE)

anova(m2)

r.squaredGLMM(m2$full_model) #Marginal and conditional Rsq for % variance explained by model

m2lmer<-lmer(EDmm~TLmm*log_ha+(1|Year),data=yp1) #use lmer() instead of mixed() for random year intercepts

ranef(m2lmer) #extract random year intercepts

#Estimated slopes for 1000 values of log_ha between data min and max

log_ha_grid<-seq(min(yp1$log_ha),max(yp1$log_ha),length.out=1000)

slopes_grid<-emtrends(m2lmer,~log_ha,var="TLmm",at=list(log_ha=log_ha_grid))

slopes_df<-as.data.frame(summary(slopes_grid))
